# Molecular pathomechanisms and cell-type-specific disease phenotypes of MELAS caused by mutant mitochondrial tRNA^Trp^

**DOI:** 10.1186/s40478-015-0227-x

**Published:** 2015-08-22

**Authors:** Hideyuki Hatakeyama, Ayako Katayama, Hirofumi Komaki, Ichizo Nishino, Yu-ichi Goto

**Affiliations:** Department of Mental Retardation and Birth Defect Research, National Institute of Neuroscience, National Center of Neurology and Psychiatry, Kodaira, Tokyo 187-8502 Japan; AMED-CREST, Japan Agency for Medical Research and Development, Chiyoda-ku, Tokyo 100-0004 Japan; Department of Child Neurology, National Center Hospital, National Center of Neurology and Psychiatry, Kodaira, Tokyo 187-8551 Japan; Department of Neuromuscular Research, National Institute of Neuroscience, National Center of Neurology and Psychiatry, Kodaira, Tokyo 187-8502 Japan; Medical Genome Center, National Center of Neurology and Psychiatry, Kodaira, Tokyo 187-8551 Japan

**Keywords:** Mitochondrial myopathy, encephalopathy, lactic acidosis, stroke-like episodes (MELAS), Mutant mitochondrial tRNA^Trp^, Mitochondrial respiratory dysfunction, Induced pluripotent stem cells (iPSCs), *in vitro* cellular disease models

## Abstract

**Introduction:**

Numerous pathogenic mutations responsible for mitochondrial diseases have been identified in mitochondrial DNA (mtDNA)-encoded tRNA genes. In most cases, however, the detailed molecular pathomechanisms and cellular pathophysiology of these mtDNA mutations —how such genetic defects determine the variation and the severity of clinical symptoms in affected individuals— remain unclear. To investigate the molecular pathomechanisms and to realize *in vitro* recapitulation of mitochondrial diseases, intracellular mutant mtDNA proportions must always be considered.

**Results:**

We found a disease-causative mutation, m.5541C>T heteroplasmy in *MT-TW* gene, in a patient exhibiting mitochondrial myopathy, encephalopathy, lactic acidosis, and stroke-like episodes (MELAS) with multiple organ involvement. We identified the intrinsic molecular pathomechanisms of m.5541C>T. This mutation firstly disturbed the translation machinery of mitochondrial tRNA^Trp^ and induced mitochondrial respiratory dysfunction, followed by severely injured mitochondrial homeostasis. We also demonstrated cell-type-specific disease phenotypes using patient-derived induced pluripotent stem cells (iPSCs) carrying ~100 % mutant m.5541C>T. Significant loss of terminally differentiated iPSC-derived neurons, but not their stem/progenitor cells, was detected most likely due to serious mitochondrial dysfunction triggered by m.5541C>T; in contrast, m.5541C>T did not apparently affect skeletal muscle development.

**Conclusions:**

Our iPSC-based disease models would be widely available for understanding the "definite" genotype-phenotype relationship of affected tissues and organs in various mitochondrial diseases caused by heteroplasmic mtDNA mutations, as well as for further drug discovery applications.

**Electronic supplementary material:**

The online version of this article (doi:10.1186/s40478-015-0227-x) contains supplementary material, which is available to authorized users.

## Introduction

Mitochondrial DNA (mtDNA) has unique translation and transcription machinery and is associated with the maintenance of cellular homeostasis through intergenomic crosstalk with nuclear DNA (nDNA). Numerous pathogenic mutations responsible for various mitochondrial diseases have been identified in mtDNA-encoded tRNA genes [1], and in most cases, such disease-causative heteroplasmic mutations (*i.e.*, wild-type mtDNA and mutant mtDNA co-exist within a cell) exhibit their intrinsic pathogenic thresholds. Mutant mitochondrial tRNAs frequently induce various loss-of-function at a molecular level [2] including not only damaged mitochondrial protein synthesis, but also inhibited aminoacylation [3], tRNA molecular instability [4], altered tRNA processing [5], wobble-base modification deficiency [6, 7], or a combination of these. Therefore, the degree of accumulated mutant mitochondrial tRNAs within a cell is most likely to determine the trajectory of tissue- and organ-specific disease progression and phenotypic severity in affected individuals. Focusing on *MT-TW* gene, which encodes mitochondrial tRNA^Trp^, several pathogenic mutations have been reported to cause widespread clinical symptoms in relation to mitochondrial diseases (*e.g.*, encephalopathy, myopathy, dementia and chorea, gastrointestinal syndrome, or severe multiple organ disorders) [8–13].

Mitochondrial myopathy, encephalopathy, lactic acidosis, and stroke-like episodes (MELAS) is genetically heterogeneous and presents a broad clinical spectrum among individuals [14] including variations in age of onset (infantile to adolescence) or affected tissues and organs (central nervous system, cardiovascular system, neuromuscular system, endocrine system, gastrointestinal system, or a combination of these). Such variations may depend on the influence of molecular defects in mitochondrial respiratory chain complexes I and/or IV (CI and/or CIV) on mitochondrial energy metabolism and oxidative stress in various terminally differentiated cell types in affected individuals. To date, however, limited somatic cell types (*e.g.*, fibroblasts, myoblasts, or lymphoblasts) are available to characterize patient-specific pathophysiology of affected tissues and organs in mitochondrial diseases. Recently, the generation of induced pluripotent stem cells (iPSCs) from various human somatic cells by forced ectopic expression of several pluripotency-associated transcription factors has been reported [15, 16], and patient-derived iPSCs carrying mutant mtDNAs have therefore opened new avenues for facilitating mitochondrial medicine.

In this study, we found a disease-causative mutation, m.5541C > T heteroplasmy in *MT-TW* gene, in a patient exhibiting MELAS with multiple organ involvement. We identified the intrinsic molecular pathomechanisms of m.5541C > T and demonstrated cell-type-specific disease phenotypes using patient-derived iPSCs carrying ~100 % mutant m.5541C > T. Our iPSC-based disease models would be widely available for understanding the definitive genotype-phenotype relationship of affected tissues and organs in various mitochondrial diseases caused by heteroplasmic mtDNA mutations, as well as for further drug discovery applications.

## Patients and methods

### Patients

This study was approved by our Institutional Review Board and was stringently conducted in accordance with the ethical principles of the "Declaration of Helsinki". Patient biopsy was performed for diagnostic purposes only after we received written informed consent with permission to study patient-derived iPSCs. Note that 10 control subjects were also used in this study.

**Fig. 1:**
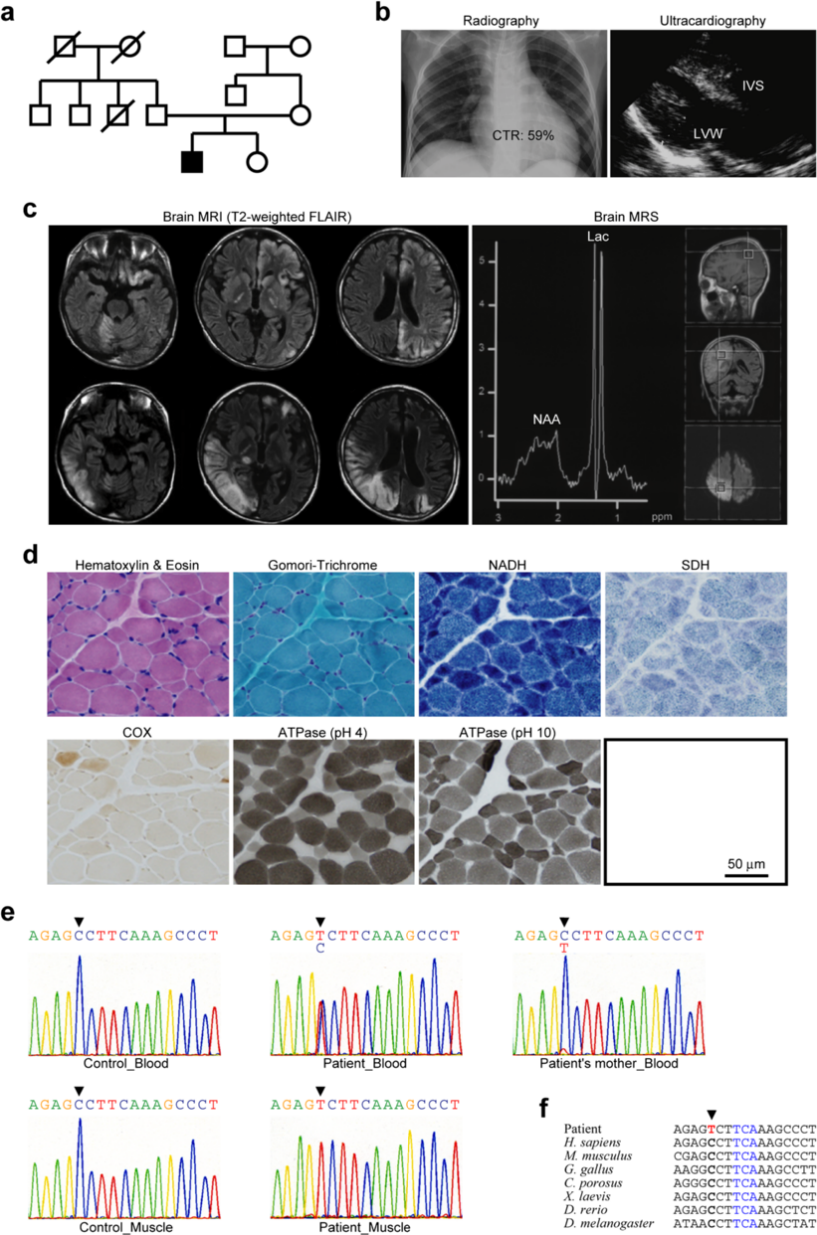
Clinical diagnosis and molecular genetic analysis for the patient. **a** A partial family pedigree. Filled square indicates this patient. **b** Radiographic and ultracardiographic images for this patient at age 11 years, indicating hypertrophic cardiomyopathy. Cardiothoracic ratio (CTR) in radiographic image is also shown. **c** T2-weighted FLAIR images of brain MRI for this patient at age 13 years, indicating multifocal stroke-like episodes. Brain MRS also shows decreased N-acetylaspartate (NAA) level and increased lactate (Lac) level. **d** Histopathology against serial frozen sections of biopsied skeletal muscle specimens from this patient at age 13 years, indicating diffuse COX deficiency. **e** Electropherograms of the anticodon domain of *MT-TW* gene for extracted DNA from blood tissues and skeletal muscle tissues of both control and this patient. Arrowheads indicate m.5541C > T. Note that the same mutation was also found in blood tissues from this patient’s asymptomatic mother. **f** Alignment of sequences in the anticodon-stem and the anticodon-loop of *MT-TW* gene from various eukaryotes. Arrowhead indicates m.5541C > T. Blue-colored characters indicate the anticodon recognition site of mitochondrial tRNA^Trp^

A partial family pedigree for this patient is shown (Fig. 1a). In this family, there was no clinical history of any neuromuscular disease. He had no growth and mental retardation until firstly presenting epileptic symptoms at age 10 years. At age 11 years, he developed weight loss, activity loss, easy fatigue, cognitive impairment, and acute heart failure. Radiographic and ultracardiographic images revealed hypertrophic cardiomyopathy at interventricular septum and left ventricular wall (Fig. 1b). Markedly increased serum lactate level (114.7 mg/dL; 3.0-17.0 mg/dL as normal), serum pyruvate level (3.86 mg/dL; 0.30–0.94 mg/dL as normal), and lactate/pyruvate ratio (29.7) were detected. At age 13 years, he developed headache, vomiting, visual disturbance, convulsion, and myoclonic status with unconsciousness. Brain MRI revealed multifocal hyper-intensity lesions at basal ganglia, cortex, and subcortical white matter of both cerebrum and cerebellum. A representative lesion showed decreased N-acetylaspartate level and increased lactate level (Fig. 1c). On this occasion, no significant abnormalities in serum lactate level (18.9 mg/dL), serum pyruvate level (0.99 mg/dL), or lactate/pyruvate ratio (19.0) were detected; however, cerebrospinal fluid lactate level (41.5 mg/dL), cerebrospinal fluid pyruvate level (1.40 mg/dL), and lactate/pyruvate ratio (29.6) were clearly high. Skeletal muscle histopathology revealed diffuse cytochrome *c* oxidase (COX) deficiency (less than 5 % population of COX-positive fibers) (Fig. 1d); however, no other typical pathological abnormalities such as ragged-red-fibers or strongly succinate dehydrogenase (SDH)-reactive blood vessels were observed. We diagnosed this patient as MELAS and started oral administration of l-arginine, dichloroacetate, and sodium pyruvate. He relapsed with stroke-like episodes twice in 4 years. At age 14 years, he developed multiple organ involvement including acute pancreatitis, gastrointestinal malabsorption, renal tubular disturbance, and endocrine glucose intolerance. At age 15 years, he developed quadriparesis. Now, he keeps stable condition without serious trouble.

### Myoblast culture

Patient-derived skeletal muscle tissues were minced, enzymatically digested, and centrifuged to collect myoblasts according to standard protocol. Patient-derived myoblasts were maintained in DMEM/F12 (Gibco) supplemented with 20 % FBS (Gibco), 100 units/mL penicillin (Gibco), 100 μg/mL streptomycin (Gibco) at 37 °C under humidified atmosphere of 5 % CO_2_. During establishment of patient-derived primary myoblasts, 0.5 μg/mL MC210 (DS Pharm) as a mycoplasmacidal reagent and 2.5 μg/mL fungizone (Gibco) as a fungicidal reagent were also added to culture medium.

Terminal differentiation of patient-derived myoblasts into myotubes was performed as follows: Briefly, patient-derived myoblasts were seeded at high cell density onto 6-well culture plates and were maintained at 37 °C under humidified atmosphere of 5 % CO_2_. After 3 days in culture, culture medium was switched to myogenic differentiation medium (Cell Applications) supplemented with 100 units/mL penicillin (Gibco), 100 μg/mL streptomycin (Gibco), and patient-derived myoblasts were maintained at 37 °C under humidified atmosphere of 5 % CO_2_ for 2 weeks.

### Analysis of mtDNA mutation

Long PCR-based whole mtDNA sequencing for the patient was performed as described elsewhere [17] with modifications to eliminate any adverse results arising from pseudo-sequences in nuclear DNA: Briefly, extracted DNA as template (10 ng for iPSCs, 20 ng for myoblasts and skeletal muscle tissues, 100 ng for blood tissues) was amplified via mtDNA-specific long-range PCR and the following mtDNA-specific nested PCR with a thermal cycler (GeneAmp PCR System 9700; Applied Biosystems). The amplified mtDNA fragments were sequenced with a DNA analyzer (ABI PRISM 3130xl; Applied Biosystems). The obtained mtDNA sequence data from each patient was compared with the databases of "Human Mitochondrial Genome Database (MITOMAP; http://www.mitomap.org/MITOMAP/)" and "Human Mitochondrial Genome Polymorphism (mtSNP; http://mtsnp.tmig.or.jp/mtsnp/)" [18] to find any genetic variants.

Mutation ratio of m.5541C > T in the patient was determined as follows: Extracted DNA (1 ng) was used as template for quantitative PCR with TaqMan Universal PCR Master Mix kit (Applied Biosystems) according to the manufacturer’s instructions. A sequence detection system (ABI PRISM 7900HT; Applied Biosystems) was used, and a calibration curve was created using several copy-number standards with plasmids containing the amplified mtDNA fragments (nucleotide position in mtDNA; 5205–5767) for either wild-type or mutant sequences.

### Protein and tRNA modeling

*in silico* modeling of bovine heart CIV homodimer in fully oxidized state [19] was performed on the database of "RCSB Protein Data Bank (http://www.rcsb.org/pdb/)", and the graphics were arranged using RasMol software. Amino acid sequences of bovine and human mtDNA-encoded CIV subunits were referenced in the database of "NCBI Protein Database (http://www.ncbi.nlm.nih.gov/protein/)".

*in silico* modeling of wild-type and mutant mitochondrial tRNA^Trp^ were performed on the database of "Vienna RNA Servers (http://rna.tbi.univie.ac.at/)", and representative physicochemical parameters (*e.g.*, minimum free energy and base-pair probability) were simultaneously calculated.

### Reverse transcription PCR (RT-PCR) and quantitative PCR

Reverse transcription was performed with PrimeScript RT Master Mix kit (TaKaRa Bio) according to the manufacturer’s instructions. After reverse transcription of extracted total RNA, total cDNA (25 ng) was used as template for RT-PCR with a thermal cycler (GeneAmp PCR system 9700; Applied Biosystems). The amplified PCR products were resolved via electrophoresis through 2 % agarose gel, stained with GelGreen (Biotium), and detected with a UV transilluminator (GelDoc-It Imaging System; UVP).

Quantitative PCR for measurement of transgenes expression was performed with SYBR Green I PCR Master Mix kit (Roche) according to the manufacturer’s instructions. After reverse transcription of extracted total RNA, total cDNA (10 ng) was used as template for quantitative PCR with a real-time PCR system (LightCycler 480II; Roche). The averaged threshold cycle number for housekeeping genes were adopted for ΔΔC_T_-based relative quantification.

### Analysis of mitochondrial enzymatic activity

Enzymatic activities for individual mitochondrial respiratory chain complexes were analyzed as described elsewhere [20] with modifications: Cultured and harvested patient-derived myoblasts (1 × 10^5^ cells/assay) were applied for measurement. A spectrophotometer equipped with thermostated unit (U-2010; Hitachi) was used, and a base line calibration was done before each measurement.

For CI activity measurement, cells were added into reaction buffer [pH 7.4; 50 mM Tris–HCl, 250 mM sucrose, 1 mM EDTA, 10 μM decylubiquinone, 50 μM NADH, 5 μg/mL antimycin A, 2 mM potassium cyanide] and were incubated at 37 °C. CI activity was monitored by time-dependent absorbance alterations.

For CII activity measurement, cells were added into reaction buffer [pH 7.4; 50 mM potassium phosphate, 20 mM succinate, 50 μM 2,6-dichlorophenolindophenol, 50 μM decylubiquinone, 5 μg/mL rotenone, 5 μg/mL antimycin A, 2 mM potassium cyanide] and were incubated at 37 °C. CII activity was monitored by time-dependent absorbance alterations.

For CIII activity measurement, cells were added into reaction buffer [pH 7.4; 50 mM Tris–HCl, 250 mM sucrose, 1 mM EDTA, 50 μM cytochrome *c*, 50 μM decylubiquinol (reduced form of decylubiquinone), 2 mM potassium cyanide] and were incubated at 37 °C. CIII activity was monitored by time-dependent absorbance alterations.

For CIV activity measurement, cells were added into reaction buffer [pH 7.4; 10 mM potassium phosphate, 25 μM ferrocytochrome *c* (reduced form of cytochrome *c*)] and were incubated at 37 °C. CIV activity was monitored by time-dependent absorbance alterations.

For citrate synthase (CS) activity measurement, cells were added into reaction buffer [pH 8.0; 125 mM Tris–HCl, 300 μM acetyl-CoA, 100 μM 5,5’-dithiobis (2-nitrobenzoic acid), 500 μM oxaloacetate] and were incubated at 37 °C. CS activity was monitored by time-dependent absorbance alterations.

### Electrophoretic separation of mitochondrial proteins

Sodium dodecyl sulfate polyacrylamide gel electrophoresis (SDS-PAGE) [21] and blue native polyacrylamide gel electrophoresis (BN-PAGE) [22] were performed as described elsewhere with modifications, respectively: Cultured and harvested patient-derived myoblasts were resuspended in isolation buffer [pH 7.4; 210 mM mannitol, 70 mM sucrose, 1 mM EGTA, 5 mM HEPES] and were homogenated on ice. Cell lysates were centrifuged to isolate mitochondrial proteins. Obtained mitochondrial proteins were quantified by Bradford assay, and a calibration curve was created using several known concentrations of BSA.

For SDS-PAGE, isolated mitochondrial proteins (100 μg) were solubilized with 0.5 % SDS containing 50 mM dithiothreitol at 70 °C for 10 min. Electrophoresis was performed on 4 –12 % NuPAGE polyacrylamide gel (Invitrogen) at room temperature under 200 V constant.

For BN-PAGE, isolated mitochondrial proteins (100 μg) were solubilized with either 0.5 % *n*-dodecyl-β-d-maltoside (individual complexes detection) or 1 % digitonin (supercomplexes detection) on ice for 30 min. Insoluble proteins were removed by centrifugation. Electrophoresis was performed on 3 –12 % NativePAGE polyacrylamide gel (Invitrogen) at 4 °C under 150 V constant for 30 min, then resumed at 4 °C under 250 V constant.

### Western blot for immunodetection of mitochondrial proteins

Electrophoresed gels were blotted onto polyvinylidene fluoride (PVDF) membranes using an iBlot transfer system (Invitrogen) according to the manufacturer’s instructions. Blotted PVDF membranes were blocked at room temperature for 30 min. Primary antibody probing was performed at room temperature for 90 min. Secondary antibody probing was performed with chromogenic antibody detection kit (WesternBreeze; Invitrogen) according to the manufacturer’s instructions. Primary antibodies used for SDS-PAGE were as follows: 0.5 μg/mL anti-porin (Molecular Probes), 2.5 μg/mL anti-MT-CO1 (Molecular Probes), 2.5 μg/mL anti-MT-CO2 (Molecular Probes), 2.5 μg/mL anti-COX4 (Molecular Probes), 2.5 μg/mL anti-COX5B (Molecular Probes). Primary antibodies used for BN-PAGE were as follows: 0.5 μg/mL anti-NDUFA9 for CI (Molecular Probes), 0.5 μg/mL anti-SDHA for CII (Molecular Probes), 0.5 μg/mL anti-UQCRC2 for CIII (Molecular Probes), 2.5 μg/mL anti-MT-CO1 for CIV (Molecular Probes), 0.5 μg/mL anti-ATP5B for CV (Molecular Probes).

### Cytochemical staining

Patient-derived myoblasts were seeded onto 4-well culture slides and were maintained at 37 °C under humidified atmosphere of 5 % CO_2_. After 3 days in culture, cytochemical staining was performed as follows:

For cytochemical COX staining, cells were stained with reaction buffer [pH 5.5; 100 mM sodium acetate, 0.1 % MnCl_2_, 0.001 % H_2_O_2_, 10 mM diaminobenzidine] at 37 °C for 1 h, followed by subsequent incubation with 1 % CuSO_4_ at 37 °C for 5 min. Cell nuclei were co-stained with hematoxylin. Stained cells were rinsed, fixed, and dehydrated according to standard histological protocol. Samples were sealed with cover glass and were observed under an optical microscope (BX50 System; Olympus).

For cytochemical SDH staining, cells were stained with reaction buffer [pH 7.4; 50 mM succinate, 1 mM nitrotetrazolium blue] at 37 °C for 1 h. Cell nuclei were co-stained with hematoxylin. Stained cells were rinsed, fixed, and dehydrated according to standard histological protocol. Samples were sealed with cover glass and were observed under an optical microscope (BX50 System; Olympus).

### Immunocytochemical detection of CIV structural subunits

Patient-derived myoblasts were seeded onto 4-well culture slides and were maintained at 37 °C under humidified atmosphere of 5 % CO_2_. After 3 days in culture, cells were fixed, permeabilized, and blocked according to standard immunocytochemical protocol. Primary antibody probing was performed at room temperature for 2 h. Secondary antibody probing was performed with 2.5 μg/mL Alexa Fluor 568 (Molecular Probes) at room temperature for 1 h. Mitochondria were co-stained with 0.25 μg/mL MitoTracker Green (Molecular Probes). Stained cells were observed under a fluorescent microscope (IX71 System; Olympus). Primary antibodies used were as follows: 2.5 μg/mL anti-MT-CO1 (Molecular Probes), 2.5 μg/mL anti-COX4 (Molecular Probes).

### Analysis of ATP level

Cultured and harvested patient-derived myoblasts (100 cells/assay) were applied for measurement. ATP amount was monitored with rLuciferase/Luciferin chemiluminescence-based ATP detection kit (Promega) according to the manufacturer’s instructions. A chemiluminescent multi-well plate reader (Centro LB 960; Berthold Technologies) was used, and a calibration curve was created using several known concentrations of ATP.

### Analysis of oxidative stress level and membrane potential level

Patient-derived myoblasts were seeded onto 96-well culture plate and were maintained at 37 °C under humidified atmosphere of 5 % CO_2_. After 3 days in culture, cells were stained at 37 °C for 1 h. Stained cells were rinsed and were measured on a fluorescent multi-well plate reader (ARVO SX; Perkin Elmer); first at excitation/emission of 545/595 nm (red fluorescence) and then sequentially at excitation/emission of 485/535 nm (green fluorescence). Fluorescent dyes used were as follows: 0.25 μg/mL MitoTracker Green (Molecular Probes), 0.25 μg/mL MitoSOX Red (Molecular Probes), 0.25 μg/mL JC-1 (Molecular Probes).

### Generation of patient-derived iPSCs with episomal vector

Patient-derived iPSCs were generated using episomal vectors as described previously [23] with modifications: Briefly, each 1 μg of episomal plasmid vectors (Plasmid #27077, #27078, #27080; Addgene) were electroporated into patient-derived myoblasts (5 × 10^5^ cells) with an electroporator (Neon; Invitrogen). Transformed patient-derived myoblasts (1 × 10^5^ cells) were reseeded onto mouse embryonic fibroblasts (MEF; ReproCELL) 4 days after electroporation. The next day, culture medium was replaced with primate ESC culture medium (ReproCELL) supplemented with 10 ng/mL bFGF (ReproCELL), 100 units/mL penicillin (Gibco), 100 μg/mL streptomycin (Gibco), and transformed patient-derived myoblasts were maintained at 37 °C under humidified atmosphere of 5 % CO_2_. Emergent colonies with ESC-like morphology were manually picked up to establish patient-derived iPSCs, and these iPSCs were expanded either on MEF-seeded dishes in primate ESC culture medium or on Geltrex (Gibco)-coated dishes in mTeSR1 medium (STEMCELL Technologies) supplemented with 100 units/mL penicillin (Gibco), 100 μg/mL streptomycin (Gibco) for long-term maintenance.

### Characterization of patient-derived iPSCs

Characterization of patient-derived iPSCs via detection of pluripotency markers was performed as follows: Briefly, cultured and harvested patient-derived iPSCs were transferred onto MEF-seeded 6-well culture plates and were maintained in primate ESC culture medium at 37 °C under humidified atmosphere of 5 % CO_2_. After 3 days in culture, patient-derived iPSCs were characterized by immunocytochemical staining. Fluorophore-conjugated primary antibodies used were as follows: 5 μg/mL Cy3-conjugated anti-OCT4 (Millipore), 5 μg/mL Cy3-conjugated anti-NANOG (Millipore), 5 μg/mL AlexaFluor 488-conjugated anti-TRA-1-60 (Millipore), 5 μg/mL AlexaFluor 488-conjugated anti-TRA-1-81 (Millipore).

*in vitro* spontaneous differentiation of patient-derived iPSCs into EB-mediated three germ layers was performed as follows: Briefly, cultured and harvested patient-derived iPSCs were transferred onto ultra-low-adherent culture dishes (HydroCell; CellSeed) and were maintained in primate ESC culture medium without bFGF at 37 °C under humidified atmosphere of 5 % CO_2_. After 7 days in floating culture, emergent EBs were transferred onto gelatin-coated 6-well culture plates and were maintained in primate ESC culture medium without bFGF at 37 °C under humidified atmosphere of 5 % CO_2_. After 14 additional days in adherent culture, spontaneously differentiated cells were characterized by immunocytochemical staining. Primary antibodies used were as follows: 5 μg/mL anti-TUJ1 for ectoderm (Abcam), 5 μg/mL anti-αSMA for mesoderm (Abcam), 5 μg/mL anti-AFP for endoderm (Abcam). Secondary antibody used was 2.5 μg/mL Alexa Fluor 568 (Molecular Probes).

### Directed differentiation of iPSCs into neural stem cells (NSCs)

Directed differentiation of patient-derived iPSCs into NSCs was performed according to a previous report [24] with modifications: Briefly, patient-derived iPSCs were seeded onto Geltrex-coated dishes and were maintained in mTeSR1 medium at 37 °C under humidified atmosphere of 5 % CO_2_. After 3 days in adherent culture, culture medium was switched to NSC induction medium [1:1 mixture of DMEM/F12 (Gibco) and Neurobasal medium (Gibco) supplemented with 1 × N2 (Gibco), 1 × B27 minus vitamin A (Gibco), 1 × GlutaMAX (Gibco), 100 units/mL penicillin (Gibco), 100 μg/mL streptomycin (Gibco), 10 μM SB431542 (Wako), 100 nM LDN193189 (Wako), 20 ng/mL EGF (Peprotech), 20 ng/mL bFGF (Peprotech)], and patient-derived iPSCs were maintained at 37 °C under humidified atmosphere of 5 % CO_2_. Emergent NSCs were expanded in NSC induction medium and were characterized by immunocytochemical staining. Fluorophore-conjugated primary antibody used was 5 μg/mL AlexaFluor 488-conjugated anti-Nestin (Millipore).

### Directed differentiation of iPSCs into neural crest cells (NCCs)

Directed differentiation of patient-derived iPSCs into NCCs was performed according to a previous report [25] with modifications: Briefly, patient-derived iPSCs were seeded onto Geltrex-coated dishes and were maintained in mTeSR1 medium at 37 °C under humidified atmosphere of 5 % CO_2_. After 3 days in adherent culture, culture medium was switched to NCC induction medium [mTeSR1 medium (STEMCELL Technologies) supplemented with 100 units/mL penicillin (Gibco), 100 μg/mL streptomycin (Gibco), 2 μM (2’Z,3’E)-6-bromoindirubin-3’-oxime (Wako), 20 μM SB431542 (Wako)], and patient-derived iPSCs were maintained at 37 °C under humidified atmosphere of 5 % CO_2_. Emergent NCCs were expanded in NCC induction medium and were characterized by immunocytochemical staining. Fluorophore-conjugated primary antibody used was 5 μg/mL FITC-conjugated anti-HNK1 (Miltenyi Biotec).

### Terminal differentiation of NSCs and NCCs into neurons

Terminal differentiation of patient-derived NSCs and NCCs into neurons was performed as follows: Briefly, patient-derived NSCs and NCCs were seeded at high cell density onto Geltrex-coated 6-well culture plates and were maintained at 37 °C under humidified atmosphere of 5 % CO_2_. After 3 days in culture, culture medium was switched to neuron induction medium [Neurobasal medium (Gibco) supplemented with 1 × N2 (Gibco), 1 × B27 minus vitamin A (Gibco), 1 × GlutaMAX (Gibco), 100 units/mL penicillin (Gibco), 100 μg/mL streptomycin (Gibco), 10 ng/mL BDNF (Peprotech), 10 ng/mL GDNF (Peprotech), 10 ng/mL NGF (Peprotech), 500 μM dbcAMP (Sigma), 200 μM ascorbic acid (Wako)], and patient-derived NSCs and NCCs were maintained at 37 °C under humidified atmosphere of 5 % CO_2_ for more than 2 weeks. For immunocytochemical detection of emergent NSC-derived neurons, 5 μg/mL anti-TUJ1 (Abcam) and 2.5 μg/mL Alexa Fluor 568 (Molecular Probes) were used. For immunocytochemical detection of emergent NCC-derived neurons, 5 μg/mL PE-conjugated anti-Peripherin (Santa Cruz) was used.

## Results

### Molecular pathomechanisms of mutant mitochondrial tRNA^Trp^

We found a patient who was clinically diagnosed as MELAS with multiple organ involvement including hypertrophic cardiomyopathy, acute pancreatitis, gastrointestinal malabsorption, renal tubular disturbance, and endocrine glucose intolerance (Fig. 1a-d). We identified a disease-causative mutation, m.5541C > T heteroplasmy in *MT-TW* gene, in this patient (Fig. 1e and Additional file 1: Table S1). Skeletal muscle tissues and the established myoblasts showed quite high mutant proportions (~100 %), whereas blood tissues showed relatively low mutant proportions (~50 %). The same m.5541C > T heteroplasmy was also observed in blood tissues from this patient’s asymptomatic mother at quite low mutation levels (~10 %). The mutated position in the anticodon-stem of *MT-TW* gene was evolutionarily conserved through most parts of primates and typical eukaryotes (Fig. 1f). We also performed *in silico* calculation of mitochondrial tRNA^Trp^ stability for both wild-type and m.5541C > T mutant (Additional file 1: Figure S1). Mutant mitochondrial tRNA^Trp^ was destabilized by m.5541C > T and was probably existed more physicochemically stable but biochemically inappropriate conformation. Our findings suggest that m.5541C > T presumably induces defects in mitochondrial tRNA^Trp^-associated translation machinery.

**Fig. 2:**
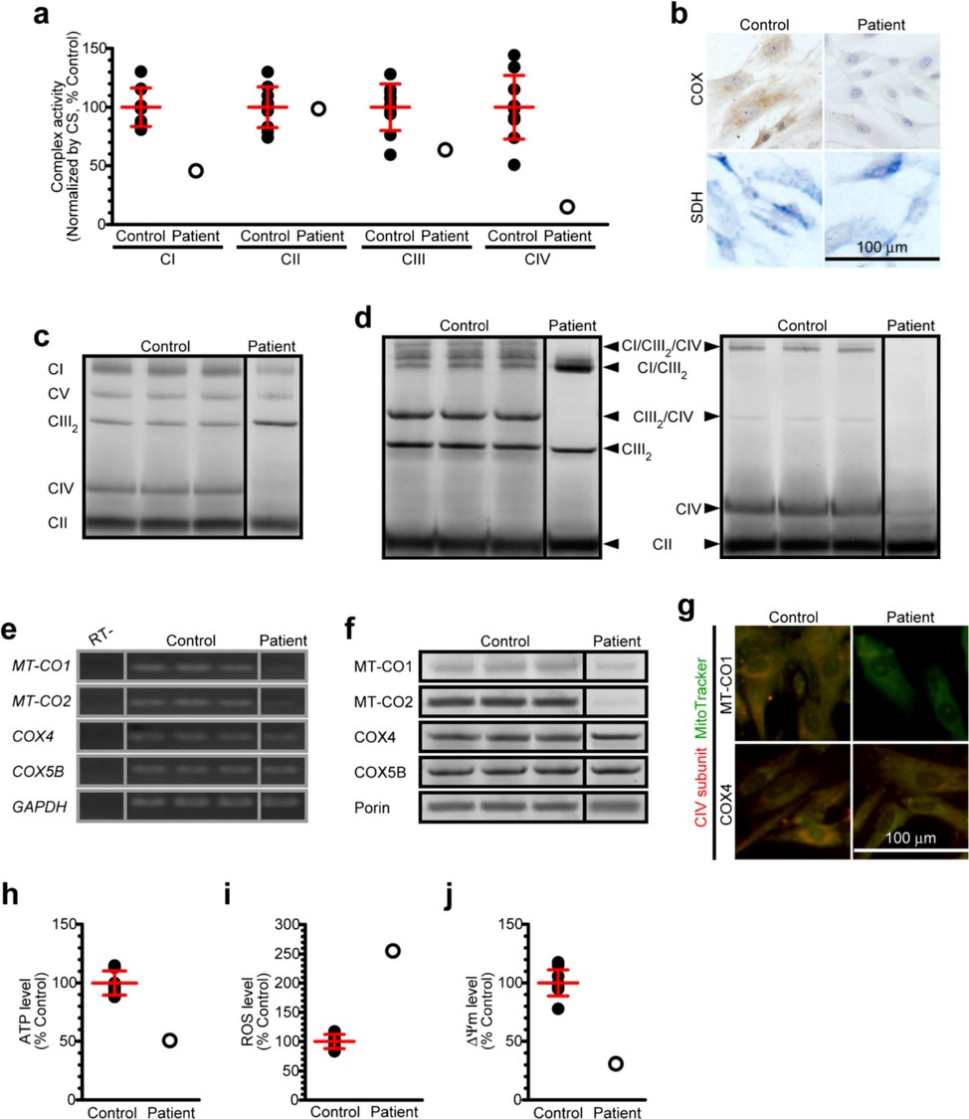
Mutant mitochondrial tRNA^Trp^ triggers widespread mitochondrial dysfunction. **a** Enzymatic activities of individual mitochondrial respiratory chain complexes for cultured myoblasts of both controls (*n* = 10, closed circles) and the patient (open circles). Error bars indicate as the means with SD of controls. All samples were measured in triplicate and averaged. **b** Representative images of cytochemical staining of COX and SDH for cultured myoblasts from both controls and the patient. Cell nuclei were co-stained with hematoxylin. **c,d** Immunodetection of **c** individual respiratory chain complexes and **d** respiratory chain supercomplexes for isolated mitochondria from cultured myoblasts of both controls and the patient. **e** Gene expression of several CIV structural subunits for extracted mRNA from cultured myoblasts of both controls and the patient. **f** Protein expression of several CIV structural subunits for isolated mitochondria from cultured myoblasts of both controls and the patient. **g** Representative images of immunocytochemical staining of CIV structural subunits for cultured myoblasts of both controls and the patient; MT-CO1 (red), COX4 (red). Mitochondria were co-stained with MitoTracker (green). **h** ATP level for cultured myoblasts of both controls (*n* = 10, closed circles) and the patient (open circles). Error bar indicates as the mean with SD of controls. All samples were measured in triplicate and averaged. **i** Oxidative stress level for cultured myoblasts of both controls (*n* = 10, closed circles) and the patient (open circle). Fluorescence intensity ratio of MitoSOX/MitoTracker served as mitochondrial ROS level. Error bar indicates as the mean with SD of controls. All samples were measured in triplicate and averaged. **j** Membrane potential (ΔΨm) level for cultured myoblasts of both controls (*n* = 10, closed circles) and the patient (open circle). Fluorescence intensity ratio of JC-1 dye aggregates/monomers served as mitochondrial ΔΨm level. Error bar indicates as the mean with SD of controls. All samples were measured in triplicate and averaged

Although m.5541C > T was previously reported and predicted as "definitely pathogenic" [26], and our patient was the second case to show this mutation, the detailed molecular pathomechanisms of m.5541C > T —how this mutation influences mitochondrial pathophysiology, which is closely related to the variation and the severity of clinical symptoms in affected individuals— remain unclear. We comprehensively evaluated mitochondrial function using patient-derived myoblasts carrying quasi-homoplasmic m.5541C > T (*i.e.*, ~100 % mutant mtDNA exists within a cell). On mitochondrial respiratory chain complexes, severely decreased CIV activity and moderately decreased CI activity were both detected in the patient, whereas the other respiratory chain complexes showed within normal ranges (Fig. 2a,b). CIV holoenzyme and CIV-containing respiratory supramolecular architectures were also diminished in the patient, whereas the other respiratory chain complexes showed almost normal levels with the exception of moderately decreased CI holoenzyme amount (Fig. 2c,d). Such CIV holoenzyme deficits in the patient were most likely because of decreased mRNA and protein expression levels of mtDNA-encoded CIV structural subunits (Fig. 2e-g). On mitochondrial physiology, widespread dysfunction such as decreased ATP level, increased oxidative stress level, and damaged membrane potential level were all observed in the patient (Fig. 2h-j). We also performed *in silico* prediction on mitochondrial tryptophan contents and their locations in each mtDNA-encoded CIV structural subunit (Additional file 1: Figure S2). Amino acid sequences in all mtDNA-encoded CIV structural subunits showed high homology between bovine and human, and bovine mitochondrial tryptophan residues were predominantly located in α-helix and β-sheet domains essential for the maintenance of CIV structural subunit conformations. Some human mitochondrial tryptophan residues in mtDNA-encoded CIV structural subunits were also located at the boundary between CIV structural subunits necessary for CIV holoenzyme assembly. Our results clearly indicate that m.5541C > T primarily induces the aberrant steady-state of mitochondrial respiratory chain complexes, followed by severely injured mitochondrial homeostasis.

### Cell-type-specific disease phenotypes of MELAS using patient-derived iPSCs carrying all mutant mitochondrial tRNA^Trp^

**Fig. 3:**
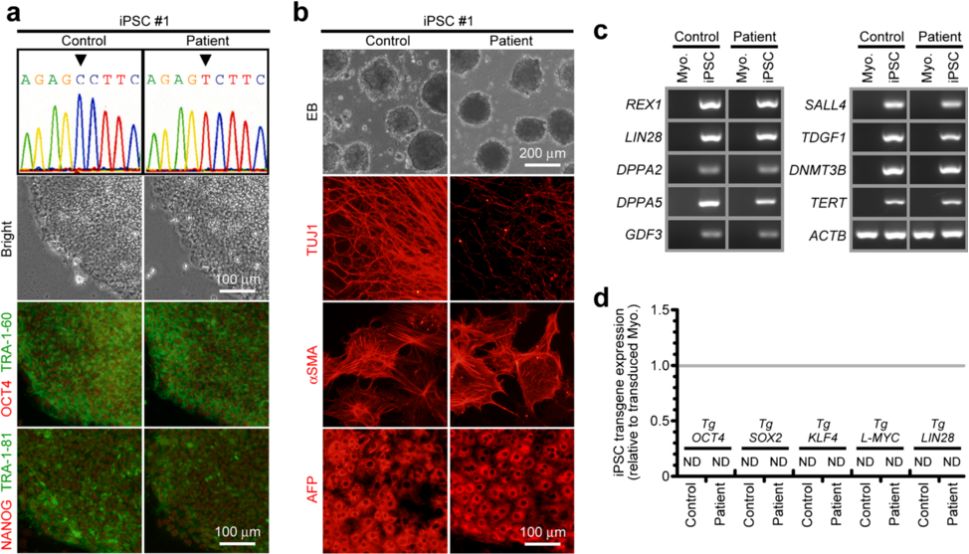
Generation of disease-relevant iPSCs carrying all mutant mitochondrial tRNA^Trp^. **a** Representative images of the established iPSC lines; OCT4 (red), NANOG (red), TRA-1-60 (green), TRA-1-81 (green). Arrowheads in electropherograms indicate m.5541C > T. **b** Representative images of the embryoid body (EB)-mediated *in vitro* spontaneous differentiation into three germ layers; TUJ1 (ectoderm, red), αSMA (mesoderm, red), AFP (endoderm, red). **c** Expression of other representative pluripotency genes in the established iPSC lines. **d** Silencing of transgenes expression in the established iPSC lines. Parental myoblasts after 7 days of transduction were also used as positive samples for both control and the patient. Expression level of each transgene was calculated using ΔΔC_T_-based relative quantification method by real-time PCR. Measurements were performed in triplicate. ND: Not Detected

**Fig. 4:**
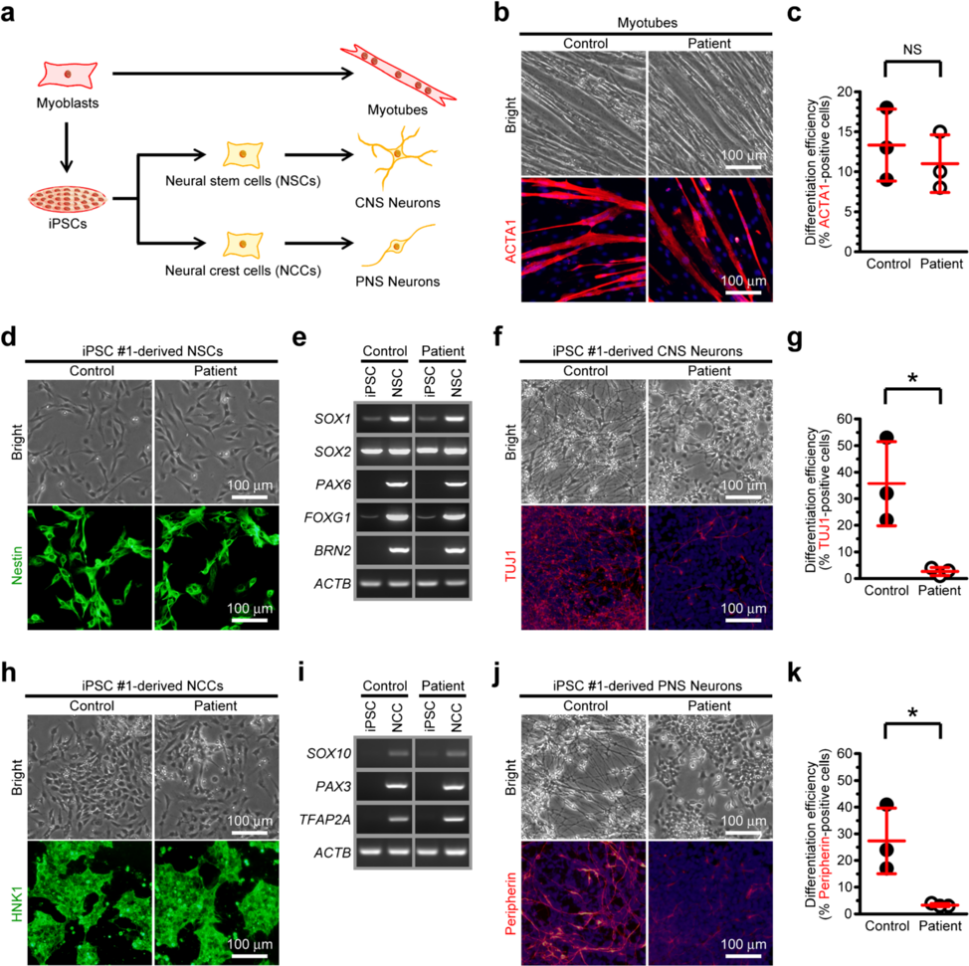
Mutant mitochondrial tRNA^Trp^ strongly impairs neuronal maturation, but does not affect skeletal muscle development. **a** Experimental design used to identify patient-specific disease phenotypes triggered by m.5541C > T. **b** Representative images of myotubes after 2 weeks of differentiation; ACTA1 (red). Cell nuclei were co-stained with Hoechst 33342 (blue). **c** Differentiation efficiency of myoblasts into myotubes. The number of ACTA1-positive myotubes was counted using a cytometer to estimate differentiation efficiency. Assays were performed using 3 experimental replicates for both control and the patient. Error bars indicate as the means with SD. Statistical significance was evaluated by unpaired, two-tailed *t*-test. NS: Not Significant. **d** Representative images of iPSC-derived NSCs; Nestin (green). **e** Expression of representative NSC marker genes in iPSC-derived NSCs. **f** Representative images of CNS neurons after 2 weeks of differentiation; TUJ1 (red). Cell nuclei were co-stained with Hoechst 33342 (blue). **g** Differentiation efficiency of NSCs into CNS neurons. The number of TUJ1-positive CNS neurons was counted using a cytometer to estimate differentiation efficiency. Assays were performed using 3 experimental replicates for both control and the patient. Error bars indicate as the means with SD. Statistical significance was evaluated by unpaired, two-tailed *t*-test. *: *P* < 0.05. **h** Representative images of iPSC-derived NCCs; HNK1 (green). **i** Expression of representative NCC marker genes in iPSC-derived NCCs. **j** Representative images of PNS neurons after 2 weeks of differentiation; Peripherin (red). Cell nuclei were co-stained with Hoechst 33342 (blue). **k** Differentiation efficiency of NCCs into PNS neurons. The number of Peripherin-positive PNS neurons was counted using a cytometer to estimate differentiation efficiency. Assays were performed using 3 experimental replicates for both control and the patient. Error bars indicate as the means with SD. Statistical significance was evaluated by unpaired, two-tailed *t*-test. *: *P* < 0.05

We next generated each 3 lines of integration-free disease-relevant iPSCs derived from myoblasts of both control and the patient carrying quasi-homoplasmic m.5541C > T as *in vitro* disease models. No apparent differences in embryonic stem cell (ESC)-like pluripotent characteristics were confirmed between iPSCs derived from control and the patient (Fig. 3 and Additional file 1: Figure S3). To elucidate patient-specific cellular disease phenotypes triggered by quasi-homoplasmic m.5541C > T, we used myotubes and iPSC-derived neurons of both control and the patient (Fig. 4a). In myogenic lineage, no significant differences in *in vitro* differentiation propensity into ACTA1-positive myotubes were observed between control and the patient (Fig. 4b,c). This phenomenon indicates that m.5541C > T seems not to affect skeletal muscle development in the patient regardless of serious mitochondrial dysfunction (see also Figs. 1d and 2). We differentiated patient-derived iPSCs into central nervous system (CNS) lineage. Efficient differentiation into nestin-positive NSCs (>95 % conversion) was observed in both control and the patient (Fig. 4d,e and Additional file 1: Figure S4a); however, the number of TUJ1-positive mature CNS neurons was markedly decreased only in the patient (Fig. 4f,g and Additional file 1: Figure S4b), and most parts of patient-derived differentiating NSCs finally died during long-term terminal differentiation (data not shown). We also differentiated patient-derived iPSCs into peripheral nervous system (PNS) lineage. A trend quite similar to CNS lineage, stable differentiation into HNK1-positive NCCs (>95 % conversion) was confirmed in both control and the patient (Fig. 4h,i and Additional file 1: Figure S4c); however, significant decrease of peripherin-positive mature PNS neurons was detected only in the patient (Fig. 4j,k and Additional file 1: Figure S4d), and patient-derived differentiating NCCs no longer survived during extended periods of neuronal maturation (data not shown). Several recent studies have demonstrated that mitochondria are gradually rejuvenated to an ESC-like "quiescent state" during cellular reprogramming [27–30]. Our results also suggest that stem/progenitor cells of both CNS and PNS lineages are minimally influenced by m.5541C > T, most likely because these cell types may possess a less active mitochondrial respiration state similar to ESCs and iPSCs. Therefore, we conclude that the molecular pathogenicity of m.5541C > T is strongly visible in terminally differentiated post-mitotic neurons, but not their stem/progenitor cells, which is probably associated with the degree of mitochondrial maturation during cellular lineage-commitment process.

## Discussion

The molecular pathomechanisms of m.5541C > T can be summarized as follows: This mutation firstly loses the appropriate base pair interaction, from Watson-Crick to T-G mismatching, in the anticodon-stem of *MT-TW* gene and induces defects in mitochondrial tRNA^Trp^-associated translation machinery most likely due to inadequate anticodon recognition of mitochondrial tryptophan by its altered conformation. Mutant mitochondrial tRNA^Trp^ disturbs the synthesis of mtDNA-encoded respiratory chain complexes subunits; in this case, markedly decreased amounts of mtDNA-encoded CIV subunits predominantly inhibit CIV holoenzyme formation at each assembly process [31]. In fact, some patients carrying this mutation or other reported pathogenic mutations in the anticodon-stem of *MT-TW* gene also present severe COX deficiency [8, 26, 32]. Therefore, our findings clearly demonstrate why mutant mitochondrial tRNA^Trp^ is able to cause severe COX deficiency as one of common clinical phenotypes. Induced mitochondrial respiratory dysfunction triggered by loss of CIV holoenzyme severely impairs mitochondrial biogenesis and bioenergetics such as decreased ATP level, increased oxidative stress level, and damaged membrane potential level. Increased oxidative stress level may promote the accumulation of oxidative damages to other mitochondrial enzymes, substrates, lipids, and mtDNA, all of which lead to premature cell senescence. Damaged membrane potential level may also accelerate the leakage of cytochrome *c* molecules in mitochondrial electron transport system, which induces apoptotic cell death. Thus, m.5541C > T causes widespread mitochondrial dysfunction, which is closely related to cell-type-specific physiological impairment in various post-mitotic tissues and organs in this patient.

This case is consistent with MELAS with multiple organ involvement characterized by its various clinical symptoms; however, we did not find any typical abnormalities in patient-derived skeletal muscle tissues other than diffuse COX deficiency. Generally, 80-90 % of MELAS patients those carrying mutant mtDNAs (*e.g.*, m.3243A > G in *MT-TL1* gene) exhibit ragged-red-fibers and/or strongly SDH-reactive blood vessels in skeletal muscle tissues, which indicate morphological, numerical, and/or physiological abnormalities in mitochondria. These findings suggest that quasi-homoplasmic m.5541C > T may cause rare pathological signatures in skeletal muscle development, presumably because this patient exhibits no significant differences in *in vitro* differentiation propensity into terminally differentiated myotubes as compared with control regardless of serious mitochondrial dysfunction.

Recently, several groups have also reported iPSC-based disease models for other heteroplasmic mutant mtDNAs [33-37]. As the common perceptions, intracellular mutant mtDNA proportions must always be considered for actual *in vitro* recapitulation of mitochondrial diseases at each cellular fate-determining process such as reprogramming, self-renewal, or differentiation. From this viewpoint, we generated integration-free patient-derived iPSCs carrying ~100 % mutant mtDNA and used them as *in vitro* cellular disease models to investigate the definitive genotype-phenotype relationship. We also demonstrated that terminally differentiated iPSC-derived CNS and PNS neurons, but not their stem/progenitor cells, are strongly influenced by m.5541C>T, most likely because the molecular pathogenic severity of mutant mitochondrial tRNA^Trp^ may be determined by the degree of physiological and morphological maturation in mitochondria. Although our presenting results do not completely elucidate the relationship between *in vitro* cellular disease phenotypes and *in vivo* clinical symptoms of this patient, our approach would be widely available for understanding *bona fide* molecular pathomechanisms and cellular pathophysiology of affected tissues and organs in patients carrying heteroplasmic mtDNA mutations, as well as for further drug discovery applications.

## Conclusions

Throughout this study, we identified the "definite" molecular pathomechanisms of m.5541C>T and demonstrated cell-type-specific *in vitro* disease phenotypes triggered by mutant mitochondrial tRNA^Trp^ using integration-free disease-relevant iPSCs derived from myoblasts of the patient. Our iPSC-based strategy therefore holds enormous promise for the development of evidence-based, personalized diagnostics and therapeutics to patients exhibiting various mitochondrial diseases.

## Additional file

Additional file 1:
**Supplementary Figures and Tables.** (DOC 4252 kb) **Figure S1.** Comparison of mitochondrial tRNATrp stability between wild-type and m.5541C > T mutant (related to Fig. 1). **Figure S2.** Protein modeling and amino acid sequences of each mtDNA-encoded CIV subunit (related to Fig. 2). **Figure S3.** Generation of disease-relevant iPSCs carrying all mutant mitochondrial tRNATrp (related to Fig. 3). **Figure S4.** Mutant mitochondrial tRNATrp strongly impairs neuronal maturation (related to Fig. 4). **Table S1.** mtDNA sequence variants in this patient. **Table S2.** Primer list. **Table S3.** TaqMan probe list.
