# Supplementary material for: Molecular pathomechanisms and cell-type-specific disease phenotypes of MELAS caused by mutant mitochondrial tRNATrp
Source: Acta Neuropathol Commun. 2015 Aug 22;3:52. doi: 10.1186/s40478-015-0227-x (PMC4546323; doi:10.1186/s40478-015-0227-x)
Supplement: Additional file 1: — Supplementary Figures and Tables. (DOC 4252 kb) Figure S1. Comparison of mitochondrial tRNATrp stability between wild-type and m.5541C > T mutant (related to Fig. 1). Figure S2. Protein modeling and amino acid sequences of each mtDNA-encoded CIV subunit (related to Fig. 2). Figure S3. Generation of disease-relevant iPSCs carrying all mutant mitochondrial tRNATrp (related to Fig. 3). Figure S4. Mutant mitochondrial tRNATrp strongly impairs neuronal maturation (related to Fig. 4). Table S1. mtDNA sequence variants in this patient. Table S2. Primer list. Table S3. TaqMan probe list. [file 40478_2015_227_MOESM1_ESM.doc]

**Supplementary Material**

**Molecular pathomechanisms and cell-type-specific disease phenotypes of MELAS**

**caused by mutant mitochondrial tRNA^Trp^**

Hideyuki Hatakeyama^1,2,*^, Ayako Katayama^3^, Hirofumi Komaki^1,3^,

Ichizo Nishino^4,5^, Yu-ichi Goto^1,2,3,5,*^

1. Department of Mental Retardation and Birth Defect Research, National Institute of Neuroscience, National Center of Neurology and Psychiatry, Tokyo 187-8502, Japan
2. AMED-CREST, Japan Agency for Medical Research and Development, Tokyo 100-0004, Japan
3. Department of Child Neurology, National Center Hospital, National Center of Neurology and Psychiatry, Tokyo 187-8551, Japan
4. Department of Neuromuscular Research, National Institute of Neuroscience, National Center of Neurology and Psychiatry, Tokyo 187-8502, Japan
5. Medical Genome Center, National Center of Neurology and Psychiatry, Tokyo 187-8551, Japan

^*^ Corresponding Author:

Hideyuki Hatakeyama, PhD (**E-mail:** hideyuki@ncnp.go.jp)

Yu-ichi Goto, MD, PhD (**E-mail:** goto@ncnp.go.jp)

Department of Mental Retardation and Birth Defect Research, National Institute of Neuroscience,

National Center of Neurology and Psychiatry (NCNP)

**Address:** 4-1-1 Ogawahigashi, Kodaira, Tokyo 187-8502, Japan

**Phone:** +81-42-346-1713; **Fax:** +81-42-346-1743


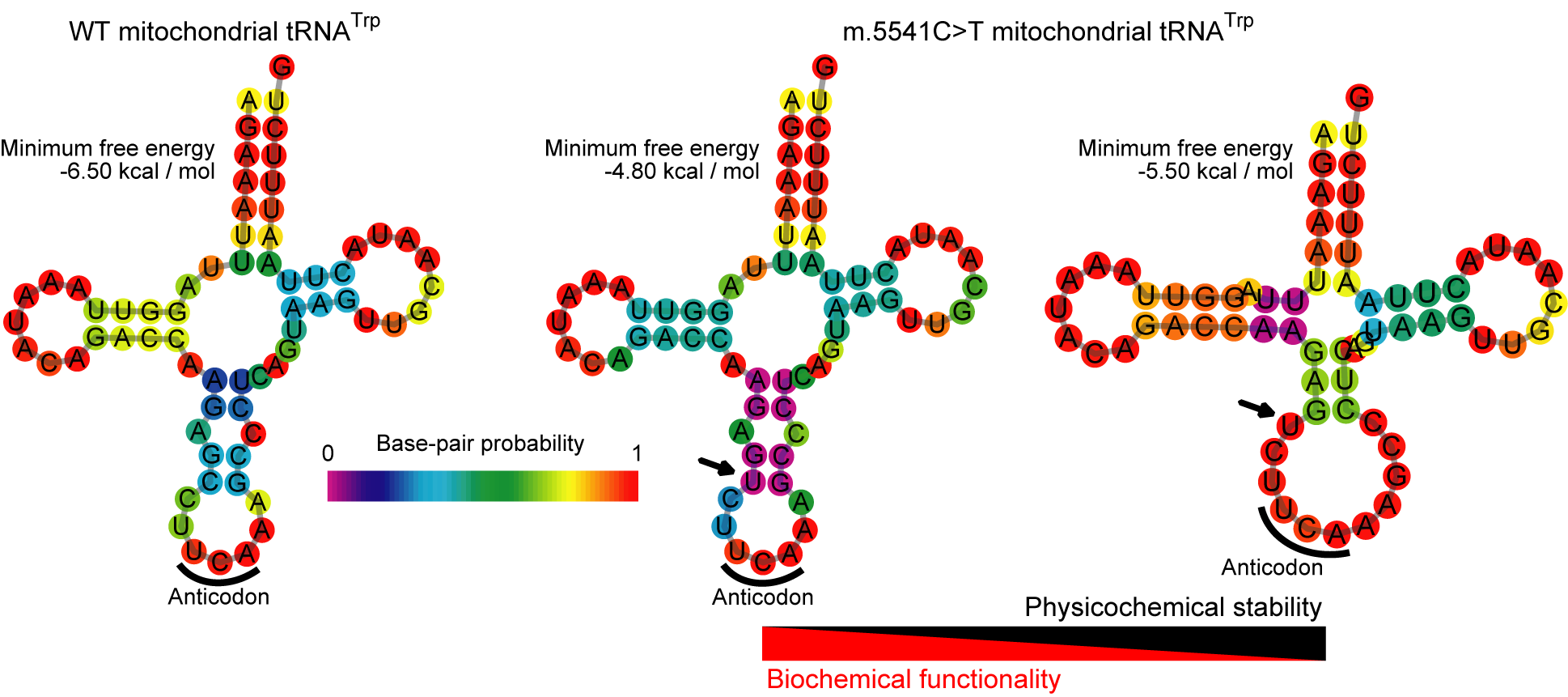


**Figure S1. Comparison of mitochondrial tRNA^Trp^ stability between wild-type and m.5541C>T mutant (related to Figure 1).**

Minimum free energies and base-pair probabilities of mitochondrial tRNA^Trp^ for both wild-type and m.5541C>T mutant were calculated on the database of "Vienna RNA Servers". Arrows indicate m.5541C>T.


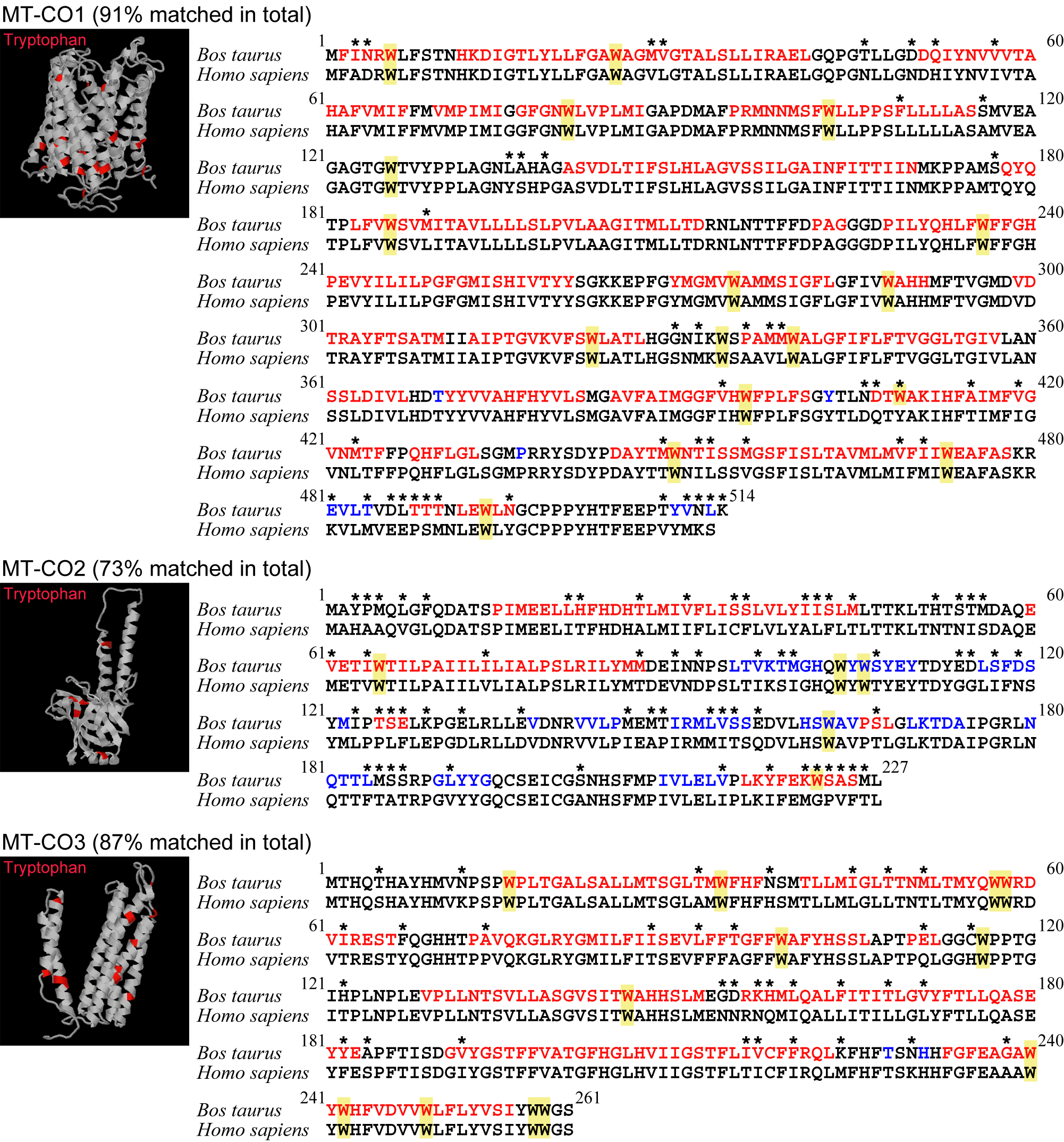


**Figure S2. Protein modeling and amino acid sequences of each mtDNA-encoded CIV subunit (related to Figure 2).**

On *Bos taurus* protein modeling, red graphics indicate the positions of mitochondrial tryptophan residues in each mtDNA-encoded CIV subunit. Data were referenced in the database of "RCSB Protein Data Bank". On *Bos taurus* amino acid sequences, red-colored characters indicate α-helix structures, blue-colored characters indicate β-sheet structures, and yellow-highlighted positions indicate mitochondrial tryptophan residues, respectively. Asterisks indicate mismatched residues between bovine and human. Data were referenced in the database of "NCBI Protein Database".


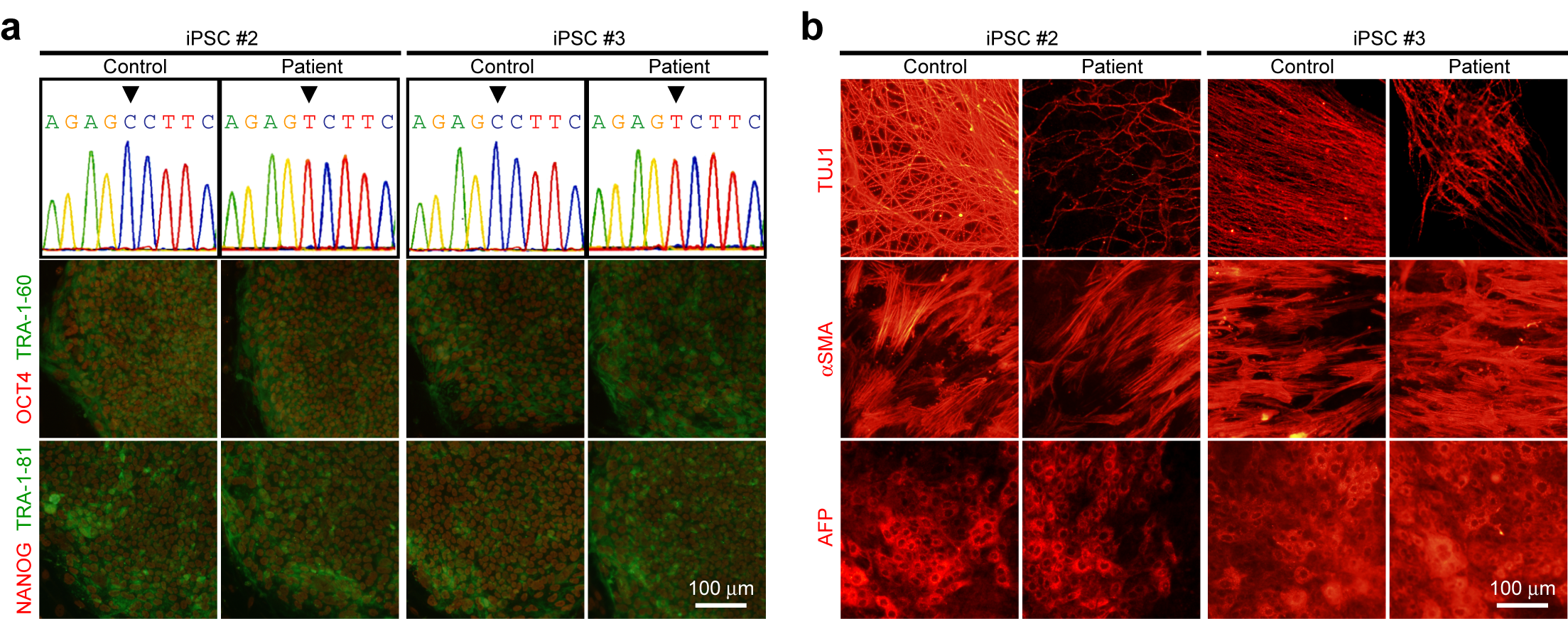


**Figure S3. Generation of disease-relevant iPSCs carrying all mutant mitochondrial tRNA^Trp^ (related to Figure 3).**

**(a)** Representative images of the established iPSC lines; OCT4 (red), NANOG (red), TRA-1-60 (green), TRA-1-81 (green). Arrowheads in electropherograms indicate m.5541C>T.

**(b)** Representative images of the embryoid body (EB)-mediated *in vitro* spontaneous differentiation into three germ layers; TUJ1 (ectoderm, red), αSMA (mesoderm, red), AFP (endoderm, red).


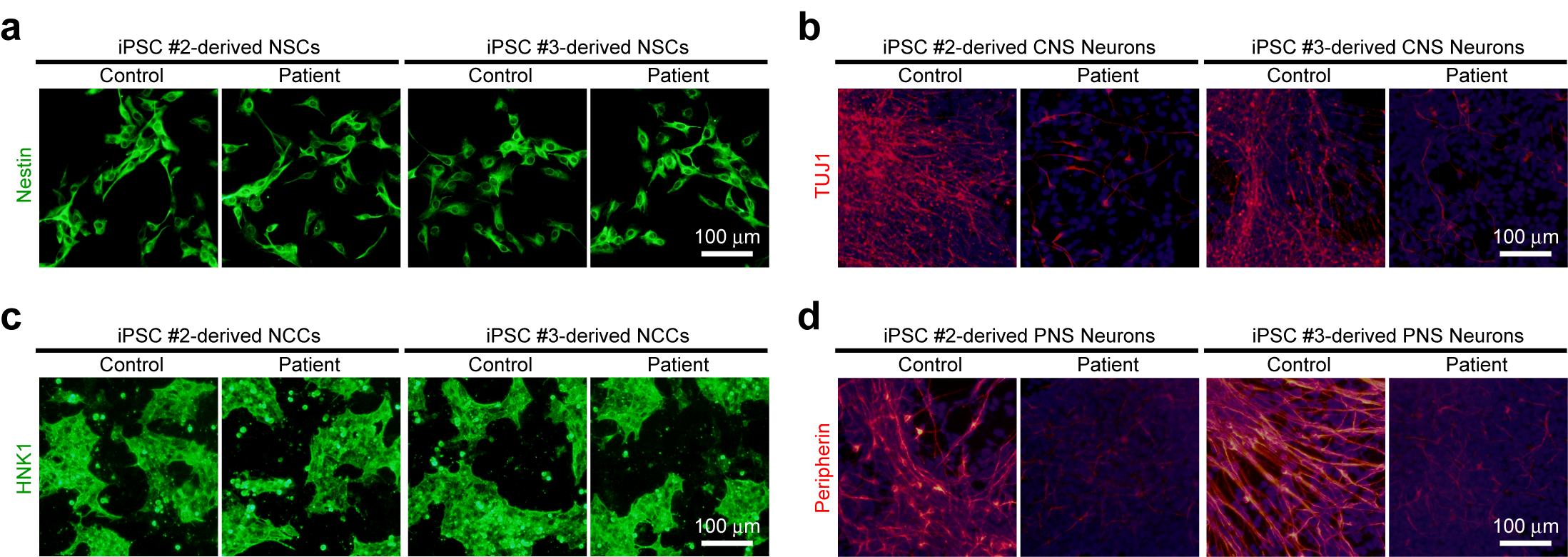


**Figure S4. Mutant mitochondrial tRNA^Trp^ strongly impairs neuronal maturation (related to Figure 4).**

**(a)** Representative images of iPSC-derived NSCs; Nestin (green).

**(b)** Representative images of CNS neurons after 2 weeks of differentiation; TUJ1 (red). Cell nuclei were co-stained with Hoechst 33342 (blue).

**(c)** Representative images of iPSC-derived NCCs; HNK1 (green).

**(d)** Representative images of PNS neurons after 2 weeks of differentiation; Peripherin (red). Cell nuclei were co-stained with Hoechst 33342 (blue).

**Table S1. mtDNA sequence variants in this patient.**

| Gene locus | Genetic variant | Amino acid change | Reported polymorphism |
| --- | --- | --- | --- |
| *MT-DLOOP* | m.73A>G |  | Reported |
| *MT-DLOOP* | m.75G>A |  | Reported |
| *MT-DLOOP* | m.89T>C |  | Reported |
| *MT-DLOOP* | m.263A>G |  | Reported |
| *MT-DLOOP* | m.303insCC |  | Reported |
| *MT-DLOOP* | m.311insC |  | Reported |
| *MT-DLOOP* | m.514delC |  | Reported |
| *MT-DLOOP* | m.515delA |  | Reported |
| *MT-RNR1* | m.709G>A |  | Reported |
| *MT-RNR1* | m.750A>G |  | Reported |
| *MT-RNR1* | m.1438A>G |  | Reported |
| *MT-RNR2* | m.2706A>G |  | Reported |
| *MT-ND2* | m.4769A>G | Synonymous | Reported |
| *MT-ND2* | m.5465T>C | Synonymous | Reported |
| *MT-TW* | m.5541C>T |  | Pathogenic (Heteroplasmy) |
| *MT-CO1* | m.7028C>T | Synonymous | Reported |
|  | m.8272-8280del |  | Reported |
| *MT-ATP6* | m.8860A>G | p.T112A | Reported |
| *MT-ATP6* | m.9123G>A | Synonymous | Reported |
| *MT-CO3* | m.9254A>G | Synonymous | Reported |
| *MT-ND3* | m.10238T>C | Synonymous | Reported |
| *MT-ND4* | m.11719G>A | Synonymous | Reported |
| *MT-CYB* | m.14766C>T | p.T7I | Reported |
| *MT-CYB* | m.15067T>C | Synonymous | Reported |
| *MT-CYB* | m.15292C>T | Synonymous | Reported |
| *MT-CYB* | m.15326A>G | p.T194A | Reported |
| *MT-DLOOP* | m.16182A>C |  | Reported |
| *MT-DLOOP* | m.16183A>C |  | Reported |
| *MT-DLOOP* | m.16189T>C |  | Reported |
| *MT-DLOOP* | m.16217T>C |  | Reported |
| *MT-DLOOP* | m.16261C>T |  | Reported |
| *MT-DLOOP* | m.16519T>C |  | Reported |

mtDNA sequence data of this patient was compared with the databases of "MITOMAP" and "mtSNP". Red-colored characters indicate m.5541C>T in the anticodon-stem of *MT-TW* gene.

**Table S2**. Primer list.

| Primer | Forward sequence | Reverse sequence | Application |
| --- | --- | --- | --- |
| *MT-CO1* | TTAGCTGACTCGCCACACTCC | AGTCAGGCCACCTACGGTGA | CIV subunit gene expression |
| *MT-CO2* | CTCATGAGCTGTCCCCACATTAG | TTGACCGTAGTATACCCCCGG |  |
| *COX4* | CGGCAGAATGTTGGCTACCA | AGCGAAAAGTCTTCGCTCTTCAC |  |
| *COX5B* | TGGCATCTGGAGGTGGTGTT | TGCCTGAAGCTCCCTTTGG |  |
| *REX1* | CAGATCCTAAACAGCTCGCAGAAT | GCGTACGCAAATTAAAGTCCAGA | Pluripotency gene expression |
| *LIN28* | TGCACCAGAGTAAGCTGCAC | CTCCTTTTGATCTGCGCTTC |  |
| *DPPA2* | TGGTGTCAACAACTCGGTTTGA | CATTTCAGGCATATCTTGCCGT |  |
| *DPPA5* | ATATCCCGCCGTGGGTGAAAGTTC | ACTCAGCCATGGACTGGAGCATCC |  |
| *GDF3* | GCTTTCTCCCAGACCAAGGTTT | CTGTTCCCTTTCTTTGATGGCA |  |
| *SALL4* | AGCACATCAACTCGGAGGAG | CCTGGGTGGTTCACTGGAG |  |
| *TDGF1* | CTGCTGCCTGAATGGGGGAACCTGC | GCCACGAGGTGCTCATCCATCACAAGG |  |
| *DNMT3B* | TGCTGCTCACAGGGCCCGATACTTC | TCCTTTCGAGCTCAGTGCACCACAAAAC |  |
| *TERT* | GCGTTTGGTGGATGATTTCT | GGCATAGCTGGAGTAGTCGC |  |
| *Tg_OCT4* | CATTCAAACTGAGGTAAGGG | TAGCGTAAAAGGAGCAACATAG | Transgene expression |
| *Tg_SOX2* | TTCACATGTCCCAGCACTACCAGA | TTTGTTTGACAGGAGCGACAAT |  |
| *Tg_KLF4* | CCACCTCGCCTTACACATGAAGA | TAGCGTAAAAGGAGCAACATAG |  |
| *Tg_L-MYC* | GGCTGAGAAGAGGATGGCTAC | TTTGTTTGACAGGAGCGACAAT |  |
| *Tg_LIN28* | AGCCATATGGTAGCCTCATGTCCGC | TAGCGTAAAAGGAGCAACATAG |  |
| *SOX1* | CACAACTCGGAGATCAGCAA | GGTACTTGTAATCCGGGTGC | NSC marker gene expression |
| *SOX2* | AACCAGCGCATGGACAGTTA | GACTTGACCACCGAACCCAT |  |
| *PAX6* | CCGAATTCTGCAGGTGTCCA | CTTTTCGCTAGCCAGGTTGC |  |
| *FOXG1* | GGCAAGGGCAACTACTGGAT | CTGAGTCAACACGGAGCTGT |  |
| *BRN2* | ACTGGGATTTACCCAAGCGG | TGCGCTGCGATCTTGTCTAT |  |
| *SOX10* | TCTGGAGGCTGCTGAACGAA | AAGTGGGCGCTCTTGTAGTG | NCC marker gene expression |
| *PAX3* | CGGCATCCTGAGCGAGCGAG | ACTCGGGCCTCGGTGAGCTT |  |
| *TFAP2A* | AGGGCCTCGGTGAGATAGTT | AAGAGTTCACCGACCTGCTG |  |
| *GAPDH* | CAATGACCCCTTCATTGACCTC | CTCGCTCCTGGAAGATGGTG | Housekeeping gene expression |
| *ACTB* | AAGATCATTGCTCCTCCTGAGC | CATACTCCTGCTTGCTGATCCA |  |
| *PPIA* | GGTCCCAAAGACAGCAGAAAAT | ACCACCCTGACACATAAACCCT |  |
| *TBP* | TTGCTGCGGTAATCATGAGG | TGGACTGTTCTTCACTCTTGGC |  |
| m.5541C>T | TGCAGTCCTTAGCTGTTACAGAAATT | GCTACTCCTACCTATCTCCCCTTTTAT | m.5541C>T mutation analysis |

**Table S3. TaqMan probe** list.

| Probe | Sequence | Application |
| --- | --- | --- |
| m.5541C>T (WT) | **VIC-**CTGAGGGCTTTGAAG**G**CTCTTGGTCTGTAT**-MGB** | m.5541C>T mutation analysis |
| m.5541C>T (Mutant) | **FAM-**CTGAGGGCTTTGAAG**A**CTCTTGGTCTGTAT**-MGB** |  |
